# Equilibrium phase diagram and thermal responses of charged DNA-virus rod-suspensions at low ionic strengths

**DOI:** 10.1038/s41598-021-82653-y

**Published:** 2021-02-10

**Authors:** Kyongok Kang

**Affiliations:** Institute of Biological Information Processing, IBI-4, Biomacromolecular Systems and Processes, Forschungszentrum Jülich, Jülich, Germany

**Keywords:** Biochemistry, Biophysics, Biotechnology, Microbiology, Biogeochemistry, Biomarkers, Chemistry, Materials science, Nanoscience and technology, Optics and photonics, Physics

## Abstract

The collective behavior of DNA is important for exploring new types of bacteria in the means of detection, which is greatly interested in the understanding of interactions between DNAs in living systems. How they self-organize themselves is a physical common phenomenon for broad ranges of thermodynamic systems. In this work, the equilibrium phase diagrams of charged chiral rods (fd viruses) at low ionic strengths (below a few mM) are provided to demonstrate both replicas of (or self-organized) twist orders and replica symmetry breaking near high concentration glass-states. By varying the ionic strengths, it appears that a critical ionic strength is obtained below 1–2 mM salt, where the twist and freezing of nematic domains diverge. Also, the microscopic relaxation is revealed by the ionic strength-dependent effective Debye screening length. At a fixed low ionic strength, the local orientations of twist are shown by two different length scales of optical pitch, in the chiral-nematic N* phase and the helical domains $H_{D}$, for low and high concentration, respectively. RSB occurs in several cases of crossing phase boundary lines in the equilibrium phase diagram of DNA-rod concentration and ionic strength, including long-time kinetic arrests in the presence of twist orders. The different pathways of PATH I, II and III are due to many-body effects of randomized orientations for charged fd rods undergoing long-range electrostatic interactions in bulk elastic medium. In addition, the thermal stability are shown for chiral pitches of the N* phase and the abnormal cooling process of a specific heat in a structural glass. Here, the concentration-driven twist-effects of charged DNA rods are explored using various experimental methods involving image-time correlation, microscopic dynamics in small angle dynamic light scattering, optical activity in second harmonic generation, and differential scanning calorimetry for the glass state.

## Introduction

Due to global warming many unknown bacteria came to existence, which can be detected by using environmental DNA from water samples^1^. The behaviors of DNA are important for exploring such new types of bacteria and detecting them. In particular, the collective behavior of DNA is an interest for the role played by the interactions between DNAs in living systems. To deal with large (or massive) numbers of statistically random sets, disregarding the stochastic or evolutionary process, in convenience, the replica method is introduced. Replica method is used such that any hierarchical structures can be represented by linking ensembles of defects in a geometric map of a discrete ultrametric space, called a Cayley tree^2^, with the “virtual” or “effective” potential barriers. Thus to predict general collective phenomena for larger set of random phases in equilibrium, any microscopic evolution theory of plastic deformation^2^ can be described by a topological “hierarchy” of an inverted tree structure of energy levels. One of the examples is typically used in spin glasses with dynamic susceptibility to decay correlations, where hierarchical defect structures form. Other examples of replica methods are also well-used for the phase diagram of weakly segregated block copolymers^3^, order parameters of the spin glasses, near the critical temperature^4^, as well as randomly distributed spins at a zero-temperature critical point^5,6^. Also, for biological systems, neuronal networks are often discussed in the stochastic process, as they are characterized by the synaptic signal changes, in the static noise input for exchanging between the synaptic strength and the anti-ferromagnetic background^7^. In the case of evolution, the energy barrier landscape is faced with expansion in multi-length scales, likewise the glasses with optimization problems in disordered and frustrated systems. Thus, it is fair to say that many uses of the replica method can be found in a wide range of natural sciences, such as mathematical taxonomy, statistical physics, complexity problems, spin glasses, neuronal networks, protein folding, and general evolutionary dynamics^8^.

The replica method is conveniently used for describing the mean-field approach of thermodynamic subsystems. Interestingly, replica symmetry breaking (RSB) occurs near the phase transitions as kinetics increase in the order-disorder and order-order transitions. So far, the most discussed systems of RSB are the mixed state of ferro- and anti-ferromagnets, as well as of the phase transitions of spin glass^9^. Unfortunately, slowly varying systems are still challenging to predict their dynamic behaviors, from molecular field theory. Instead, mostly static pictures of possible ensemble averages are accessible with the nonergodicity for given lengths and times. To overcome such gaps existing between pure theory and real mechanisms, in this article, a model system of charged rods is employed for the interests of thermodynamic subsystems, which are specifically as follows: (i) first, the phase boundaries formed between the structural glasses and other chiral mesophases, as well as the long-time kinetic arrest (LTKA) are discussed in the equilibrium phase diagram of charged chiral DNA virus fd rod-concentration versus ionic strength. By lowering the ionic strength, extended regions of the chiral nematic N* phase are observed with hierarchical chiral mesophases (of X-pattern and helical domains). These chiral mesophases undergo extremely slowed relaxation and form structural glasses, found at high DNA-rod concentrations and the low ionic strength of 0.16–0.8 mM salt below the critical ionic strength (of 1–2 mM). (ii) The second interest of the subsystem is the helicity that appears in the chiral-nematic N* phase and chiral mesophases (of X-pattern and helical domains) with “twist” elasticity. Helicity is a genuine feature of the broken symmetries, either from the geometry of a single particle or the topological twist of a collection of small particles. The chiral nature of orientation textures is easily seen in many biological systems with their motility to serve the biomechanical properties, forming twisted rigid rods (by mobile ions)^10^ and bundles of filaments in chromosomes^11^. Different geometrical structures are seen as toroidal DNA condensates^12,13^ due to the elastic deformations in DNA fragments^14^. The twist also plays a role, both in the intrinsic twist of fibrinogen molecules^15^ and the variation of helical twist power exhibited by chiral rod-coil amphiphiles^16^. Thus, the self-assembly of natural chiral rods naturally buildup of complex structures with biochemical functional groups relating to the synthetic fibers and rods. However, there is rare evidence in experiments for metastable helical arrangements in the 3D bulk state for long-range disorder-order transitions. Here, the suspensions of highly charged DNA viruses (fd) at low ionic strengths are shown as a good model system for stable chiral mesophases in a bulk equilibrium state. (iii) Third, replica symmetry breaking (RSB) is present in the equilibrium phase diagram of orientation textures in the suspensions of charged DNA viruses (fd) at various low ionic strengths: in the replica, self-similarity of optical pitches is observed in different length scales, as larger in the N* chiral-nematic phase (at low-concentration) and shorter in tightly packed helical domains (at high-concentration). Also, in between them, the X-pattern is observed (in medium concentration) as the RSB broadly at low ionic strengths. This is because the effective interaction varies with the ionic strength; at a higher ionic strength, above the critical ionic strength, relatively fast relaxations occur in the stable planar-nematic. In contrast, just below the critical ionic strength, the microscopic dynamics of structural glass are much slower, coexisting with the freezing of nematic-domains. Such different microscopic dynamics resulting from varying the ionic strength for a given DNA virus suspension affects to the collective thermal fluctuations, sufficiently coupled to their different preferred orientations. Therefore, the aim of this paper is to show the conclusive equilibrium phase behaviors of charged DNA virus fd rods at low ionic strength, where the electric-double layer dependent rotational motions respond collectively to the “twist” of charged rods^17^. Few locations of long-time kinetic arrests (LTKAs) are analyzed as different pathways in the phase diagram near the structural glass via image-time correlation to quantify the decays of long-lived orientation textures. In addition, thermal responses of the system are checked for the reversible twist effect of chiral-nematic pitches, as well as their abnormal specific heats in the charged rod glass.

**Figure 1:**
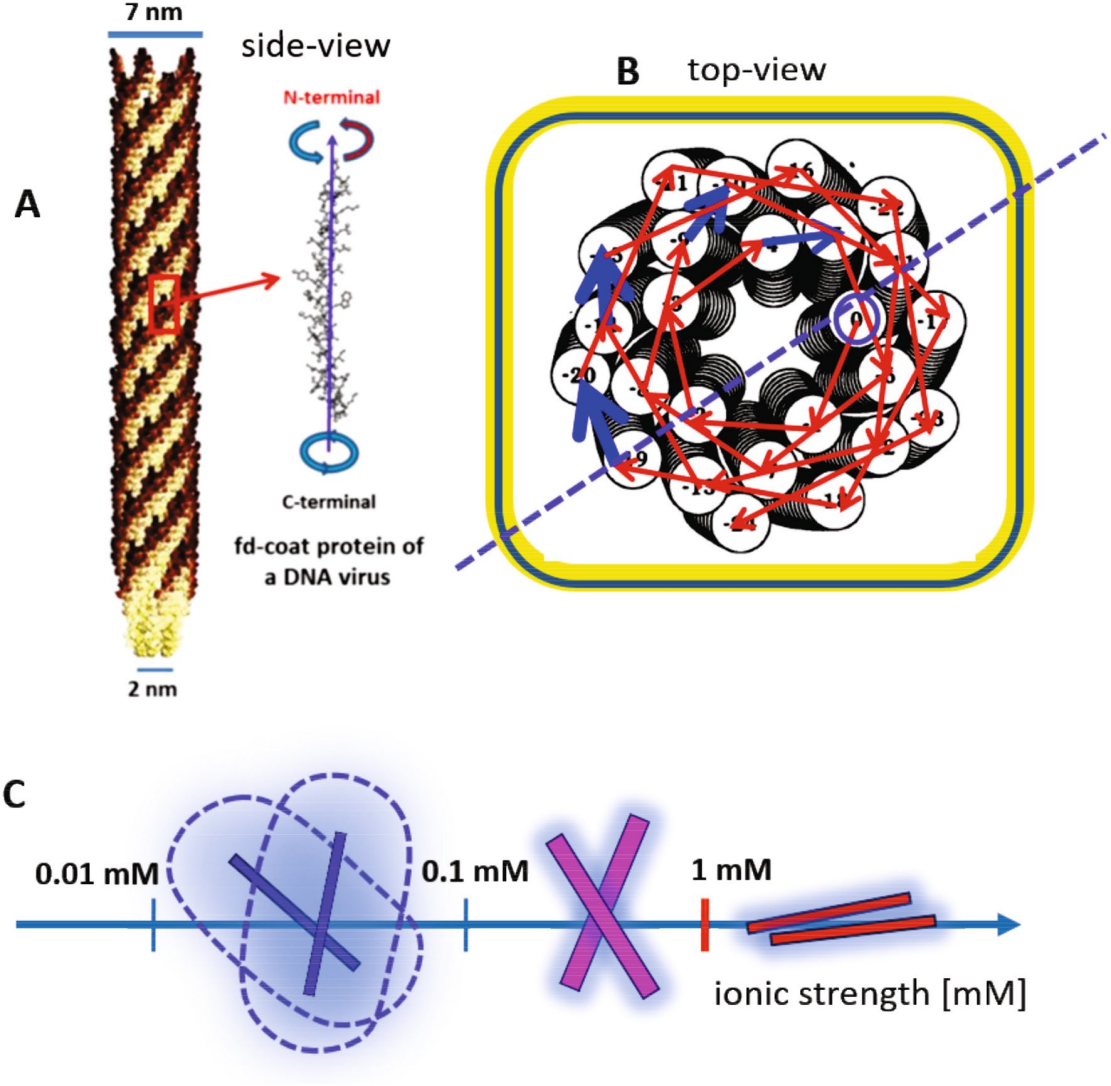
The molecular structure and effective arrangements of charged DNA viruses: the side-view of bacteriophage fd with its left-handed chiral coat proteins that are surrounded along the long axis is shown in (**A**). The fd coat protein consists of 50 amino acid terminals, ending with an open N-terminal and the closed C-terminal. The top-view of the fd coat protein is shown in (**B**), where the open N-terminal is shown with fivefold asymmetric layers: The red arrows indicate the consecutive links of the sites and the thick blue arrows indicate the last terminal site of each layer to open the next outer layer. A simple guide of the effective arrangement for two interacting charged DNA rods is illustrated in (**C**), as the ionic strength increases above the isotropic-nematic coexistence concentration.

## Charged DNA-virus rod suspensions

A bacteriophage fd is a type I inovirus DNA strand. It is well known that the conformations of inoviruses can be varied, from $\alpha$-helix to $\beta$-sheet, depending on the pH values and ionic strengths of the environments. A self-assembled DNA-virus rod consists of many surrounding fd-coat proteins that total 50 amino acids of an alpha-helix (see Table 1 in ref.^18^), as shown in Fig. 1A. Interestingly, the ends of fd-coat proteins are composed of two different terminal sites; one is the open and slightly screwed loop of the amine N-terminal, and the other end is the closed carboxylate C-terminal, depicted in Fig. 1A,B. The top-view in Fig. 1B is labeled to better describe the terminal site links of each layer, taken from the Ref.^19^: The open N-terminal of the fd-coat proteins is connected to form a few layers of a fivefold “open-loop”, and the 6th site is slightly skewed along the fd-viron axis connected to the start of the next layer (see the arrows in Fig. 1B). The single molecular structure of a filamentous bacteriophage fd is a long ($L=880$ nm) and thin (a bare diameter of $D=6.6$ nm) rod-shaped DNA strand with a left-handed chiral core^20,21^. The number of thymine–adenine (T–A) pairs of the fd virus is found to be higher than that of the guanine–cytosine (G–C) pairs in the DNA base. The persistent length of an fd virus particle is about 2200–2800 nm, and the surface is surrounded with 2700 fd-coat proteins twisted in a helical structure along the filament axis^22^. The total charges of an fd virus are estimated to be 8800, and 7500 negative elementary charges for a low ionic strength of 0.16 mM and 0.032 mM respectively^23^. The detailed description of the preparation of DNA virus fd rod suspensions given here is the same as that provided in Ref.^24^, which includes the effect of dissolved carbon dioxide on both ionic strength and *pH*. As compared to previous works of higher ionic strength (above 1 mM salt), the effective aspect ratio, defined as $L/(D+\kappa ^{-1})$, is reduced to as 26 and 17, for a low ionic strength of 0.16 mM and 0.032 mM respectively. The effective aspect ratio is then decreased by lowering the ionic strength, with an increased Debye length of $\kappa ^{-1}$, due to the dissociation/association of condensed ions that are surrounded with the core of fd viruses to the bulk salt solution.

Theoretically, Onsager showed that the location of isotropic-nematic binodal and spinodal concentrations depends on the dimensionless concentration of $(L/d)\,\varphi$, where *L* is the length of the rod, *d* the core diameter, and $\varphi = (\pi /4)\,d^{2}\,L\,\rho$ the volume fraction, and $\rho$ as the number concentration of rods^25^. The Onsager theory are still valid even in the case of charged-rods by replacing an effective diameter $d_{ef\!f}$ and effective dimensionless concentration $(L/d_{ef\!f})\,\varphi _{ef\!f}$. If the particle has a high anisotropic ratio, the system undergoes an isotropic–nematic (I–N) phase transition, depending on the ionic strength^24,26^. Thus the isotropic-nematic (I+N) coexistence (as a biphasic phase) concentration at a lower ionic strength occurs at a lower DNA rod concentration, shown effectively by the twist effect becoming stronger as the concentration increases, resulting in a shorter pitch length. Also, by decreasing the ionic strength, the twist becomes stronger due to the increased Debye screening length, as well as becoming perpendicular to each other, as illustrated in Fig. 1C. Thick electric double layers surrounding the charged DNA viruses render the “twist” elasticity, allowing for the enhanced perpendicular alignments of two interacting rods below the ionic strength of 1 mM. In contrast, above 1 mM, parallel alignment is dominant for thin double layers. In the present case, where the Debye length to the core diameter is varied, the effective diameter can be expressed as (see Refs.^27,28^),

1 $$\begin{aligned} d_{ef\!f}\;=\;\kappa ^{-1}\,\left[ \,\ln K_{Q}+\gamma _{E}\,\right] \;, \end{aligned}$$

where $\kappa ^{-1}$ is the Debye length and $\gamma _{E}=0.5772\cdots$ is Euler’s constant. Here the special interest is the degree of dissociation of condensed ions, as

2 $$\begin{aligned} K_{Q}= & {} \frac{4\,Z^{2}\,\exp \{\kappa \,d\}}{(\,1+\kappa \,a)^{2}}\,\frac{l_{B}}{\kappa \,L^{2}}\;, \end{aligned}$$

with *Z* the valency of the entire rod, and $l_{B}=0.75$ nm as the Bjerrum length.

A series of studies has been performed on bacteriophage DNA-viruses (fd), both in non-equilibrium^29^ and equilibrium conditions^17,24,30^. These systems serve as colloidal model systems of charged rods, which allow to study their collective behavior and to develop (semi-empirical) theories for their orientational dynamics^28,29^, field-induced criticality^31^, and critical slowing down of dynamics near phase boundaries^27,31^. These studies go well beyond the classic Onsager theory for the isotropic–nematic transition, where the chiral nature of the molecular structure of the DNA viruses lead to a twist energy that in turn leads to the formation of a chiral-nematic phase as well as several hierarchical chiral-mesophases on increasing the virus concentration^17^.

**Figure 2:**
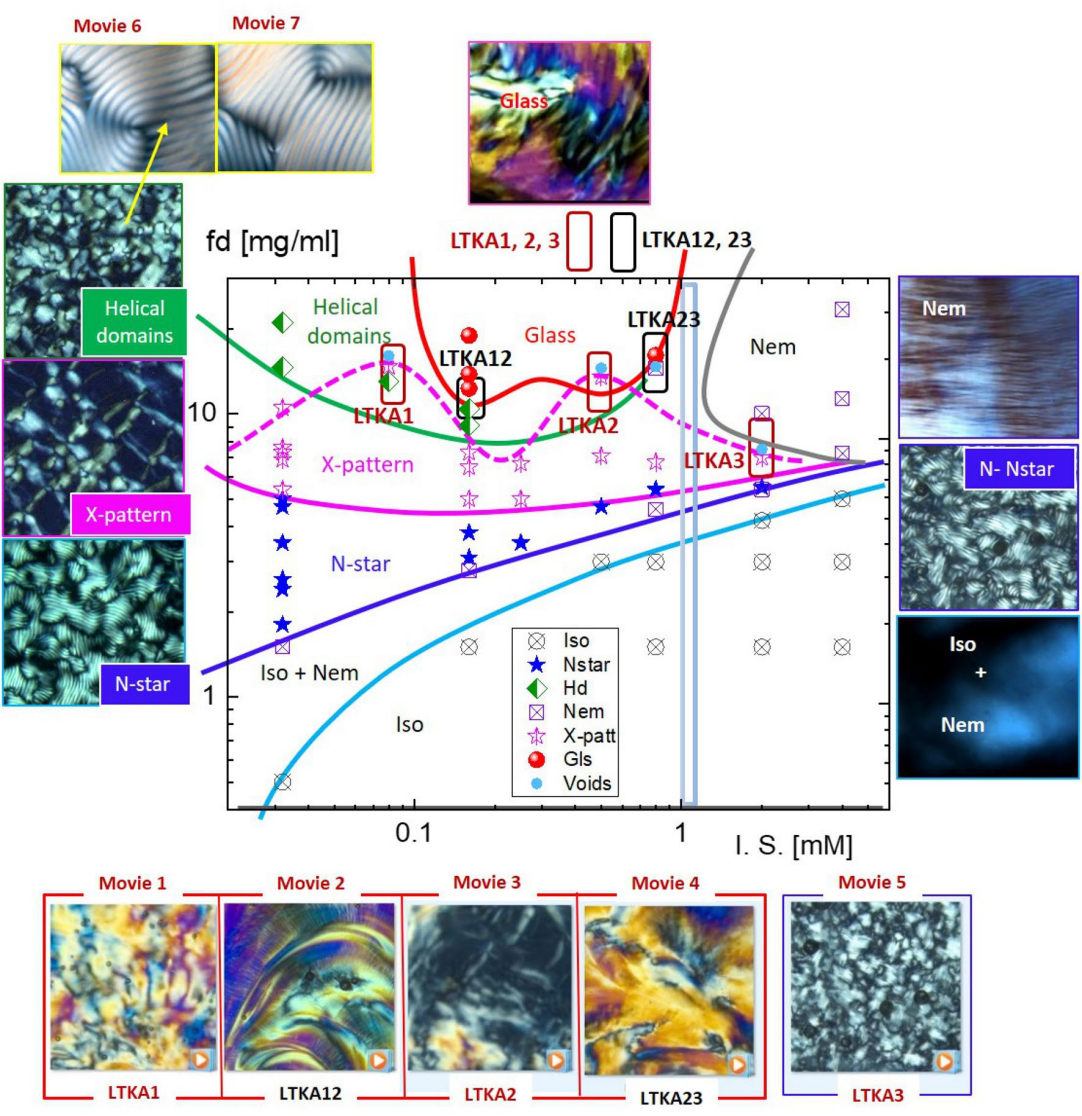
The equilibrium phase diagram and the corresponding optical morphologies of charged fibrous viruses (fd) as a function of fd-concentration and ionic strength (below 4 mM salt): The various phases are indicated as Iso: isotropic, Iso + Nem: isotropic-nematic coexistence, N-star: chiral-nematic (N*), X-pattern, helical domains ($H_{D}$), and glass. The phase lines are guided for each observed phase data point. Near the glass, the fluctuating X-pattern exists with mixed states of helical domains and occasional “voids”. A few locations of long-time kinetic arrest (LTKA) are indicted as LTKA1, LTKA2, LTKA3, LTKA12, and LTKA23 near high concentrations that exceed the phase boundary in the diagram. Note that LTKA3 is located at a lower concentration, compared to other LTKAs at higher ionic strength. Supplementary data movies are provided in Movies 1, 2, 3, 4 and 5 for LTKAs, as well Movies 6 and 7 for existing slow dynamics of microscopic views of helical domains respectively, via ImageJ software version ImageJ 1.52i in the URL link https://imagej.nih.gov/ij/.

Thus the reason for much slowing down behavior in microscopic dynamics is the forming of collective soft-modes for low ionic strengths in the chiral-nematic phase, as confirmed by dynamic light scattering^17^. This then leads to the glass concentration, reached at a higher concentration, for the particularly low ionic strengths (of 0.16–0.8 mM salt). Below the glass transition concentration, different orientation textures are equilibriated for a long waiting time (of 80–100 h)^30,32^. One should note that the driving mechanism of 3D chiral-nematic orientation textures in this work is completely different to the director field by the curvature space and anchored force fields for small cholesteric liquid crystal molecules. The observation here is mainly by the charged DNA rod-rod interactions that occur when the critical ionic strength (of 1–2 mM salt) is exceeded, between the short-range direct interaction and the long-range hydrodynamic interaction. These DNA rod-rod interactions are presented in the observed equilibrium phase diagram, in Fig. 2, for the stable and meta-stable states (LTKAs) of bulk phases. More features of several phases are discussed in the following sections, including asymmetric phase boundary lines that appear in the N–N* transition (for low ionic-strength), X-pattern, helical domains, and the few LTKAs, as well as the planar-N phase (for high-ionic strength) above the critical ionic strength.

## Equilibrium phase diagram of DNA-virus rod-concentration and the low ionic strength

Before the discussion on equilibrium phase behaviors, electric field-induced nonequilibrium phenomena have been observed in DNA-virus suspensions at low ionic strengths^28,29^. Electric fields can induce phases and dynamical states for concentrations within the isotropic-nematic two-phase region. For low frequencies the electric field induces a transition where nematic domains become chiral nematic, and a dynamical state where the domains persistently melt and reform. However, for sufficiently high frequencies, charge polarization does no longer occur, a transition is observed from nematic–isotropic coexistence to a homogeneous homeotropic state. In addition, there is a non-equilibrium critical point, in the sense that a time scale (on which melting and reforming of domains occurs) and a length scale (the size of helical domains) diverge^28,31^, mimicking the similarity of a critical phenomenon in the equilibrium dynamics, originated by the dissociation and association of condensed ions on the surface of DNA viruses.

In addition to a complex response to electric fields, there is also a quite interesting response of the fd-virus suspensions to an externally imposed flow at relatively high concentrations and low ionic strengths^33^, where a structural glass is observed^30,32^. Here, structural arrest is due to long-ranged electrostatic interactions, leading to a soft Wigner glass. Several non-uniform 3D flow profiles are observed, depending on the concentration and the applied shear rate: fractured flows, gradient shear-banded flow profiles, and vorticity-banded flow profiles.

In the current work, we explore the phase behavior and kinetics of concentrated DNA fd-virus suspensions at various low ionic strengths, which has not been explored before, and turns out to be quite different from the behavior at high ionic strengths.

**Figure 3:**
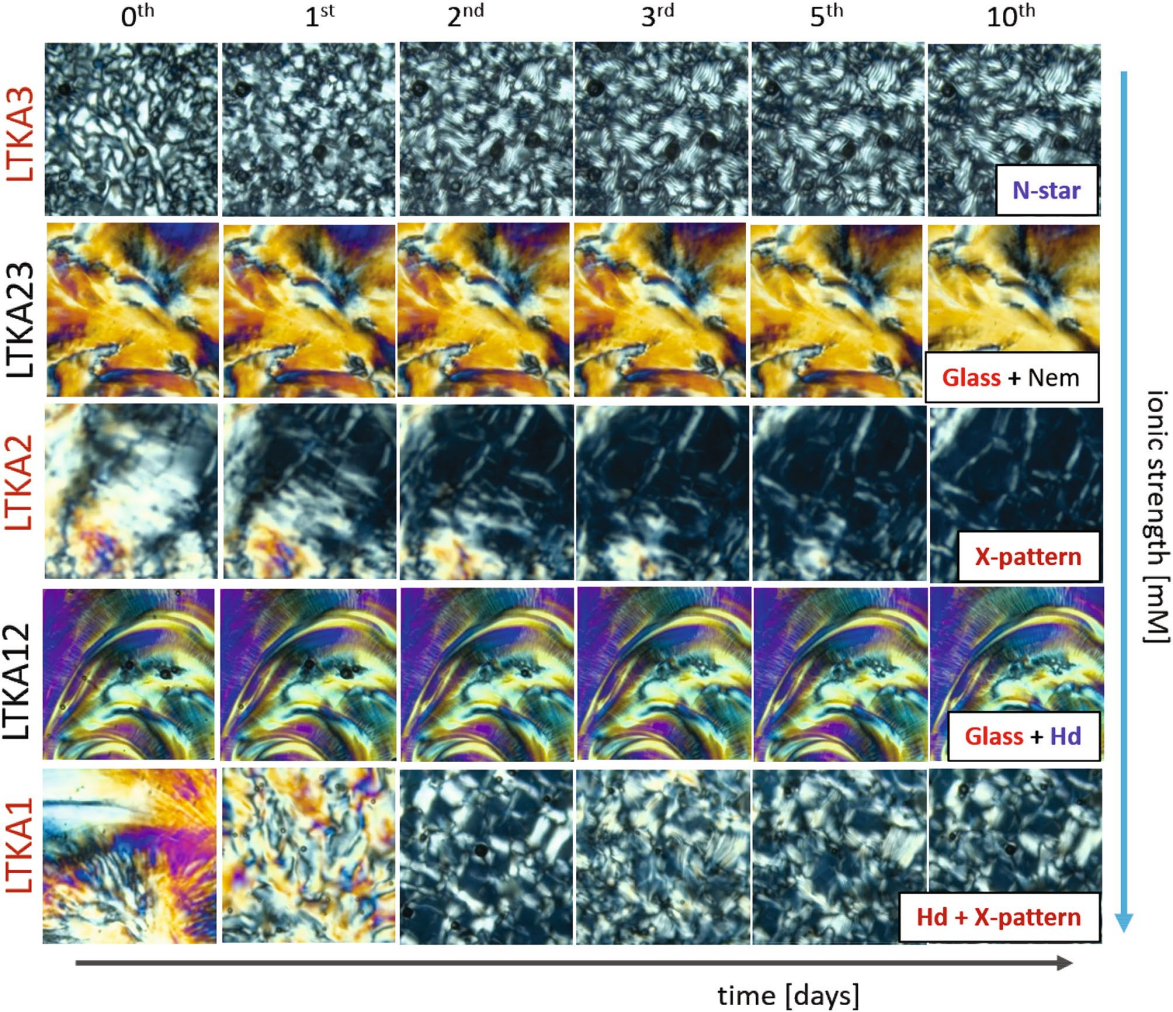
The long-time temporal morphologies of LTKAs in the equilibrium phase diagram: Long-time kinetic arrests are even distinguished by their rate of change in the optical morphologies, which are faster in LTKA1, LTKA2, and LTKA3, while being much slower in LTKA12, and LTKA23. The final states of the LTKAs are also only shown as clear X-patterns (in LTKA2) and the X-pattern with helical domains (in LTKA1), as well as with partial “voids”. The appearance of “voids” tends to be arbitrary in these LTKAs in the diagram. Note that LTKA3 is of a lower concentration and higher ionic strength than the other LTKAs. A software version ImageJ 1.52i is used in the URL link https://imagej.nih.gov/ij/.

**Figure 4:**
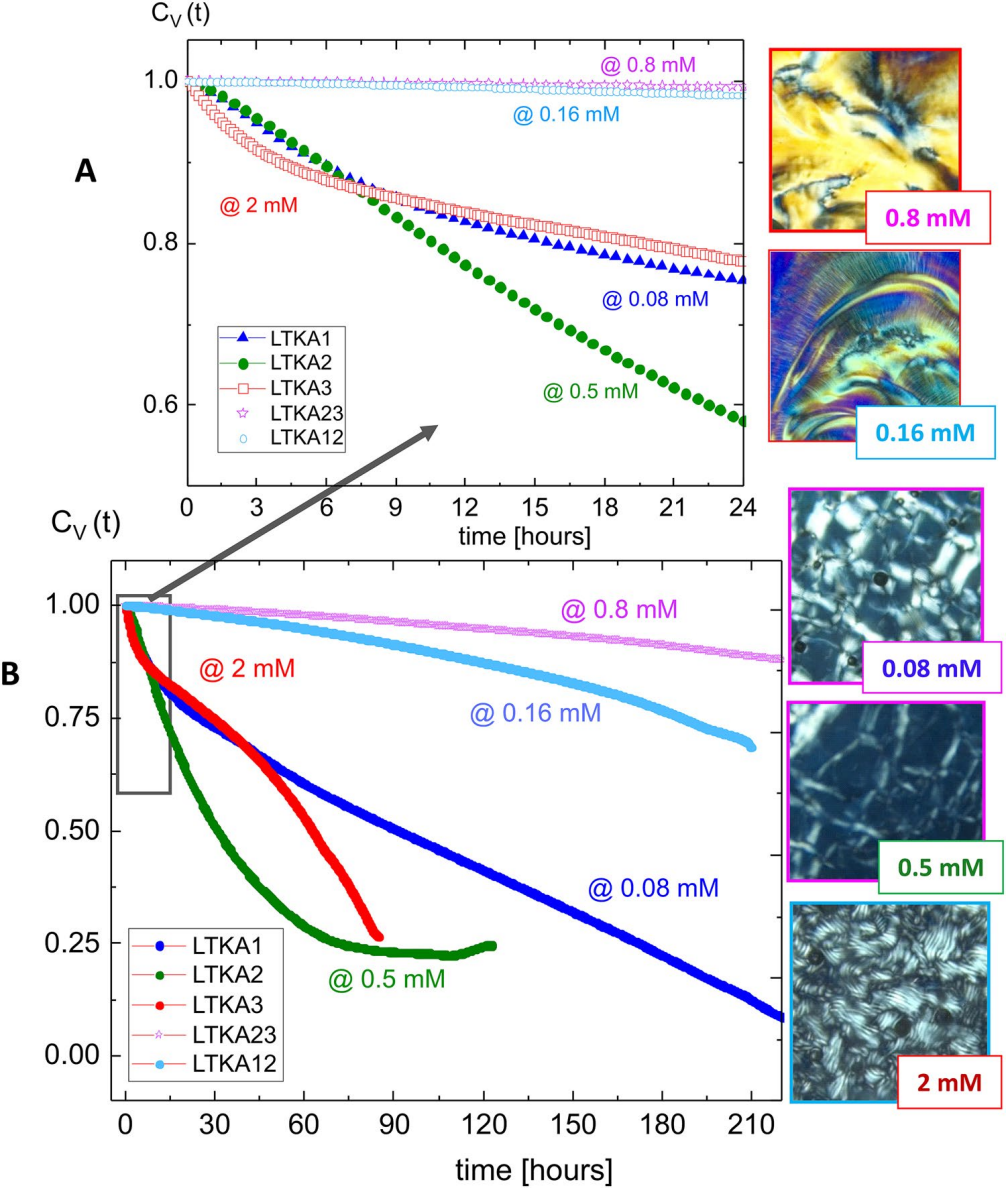
The results of long-time kinetic arrests (LTKAs), obtained by image-time correlation and the final state of the equilibrium phase for varying the ionic strengths: (**A**) for a shorter time duration, and (**B**) for longer-time correlations to reach the corresponding phases, which are the glass (in 0.16–0.8 mM), X-pattern (in 0.5 mM), the mixed state of X-pattern and the helical domains (in 0.08 mM), and N–N* (N-star) (in 2 mM). The results of image-time correlations for the orientation textures are shown as a function of ionic strengths. Slow decays are carried out for two ionic strengths in the glass state, in LTKA12 and LTKA23. According to the result, LTKA23 turned out to slow down even faster than LTKA12. However, near the glass, the X-pattern exists with mixed states of the helical domains (in 0.08 mM) and occasional voids. LTKA1, LTKA2, and LTKA3 are all decayed to below a value of 0.25 in correlations. Note here that concentrations of above 4 in ionic strength are higher than in the case of 2 mM, thus it also decays faster compared to the others.

The equilibrium phase behaviors of anisotropic-shaped particles are interesting for understanding different interactions of orientations with their effective (and collective) alignments. Especially for a rod-like shape, the isotropic–nematic (I–N) coexistence phase is varied by the physical parameters of its aspect ratio (or the length/diameter), as well as the degree of orientation order (known as order-parameter). Furthermore, for charged rods, the I–N transition is influenced by the amount of dissociation of condensed ions into mobile ions, which form significantly larger electric double layers surrounding the DNA-virus. This not only lowers the aspect ratio, resulting in an increase of the effective diameter with a larger Debye screening length, but also introduces news chiral mesophases with boundaries in the phase diagram. To illustrate the main features of equilibrium phase behaviors, the dynamic pathways of RSB are summarized in the phase diagram of charged rod-concentration versus ionic strength in Fig. 2: As the ionic strength decreases, the nematic phase becomes unstable compared to the chiral-mesophase (of X-pattern) and chiral-nematic N* phase. The main features of the equilibrium phase diagram (in Fig. 2) for charged DNA virus fd rods are summarized as follows: Over the course of variations ionic strength, the LTKAs are illustrated with movie data sets using image-time correlation spectroscopy. The critical ionic strength of greater than 1 mM is also indicated as a vertical line in the phase diagram, where LTKA23 and LTKA3 are shown. The appearance of “voids” is rather arbitrary but present.

(I) A sharp (and global) crossover transition occurs at an ionic strength of 1–2 mM salt in Fig. 2: Below 1 mM, a stable chiral-nematic N* phase and chiral-mesophases (X-patterns and the helical domains) are formed, however, at higher ionic strengths, only the planar-nematic phases form. It turns out that the collective motions of relaxing DNA rods are affected by their perpendicular motions, probed by depolarized dynamic light scattering (see the “Long-time kinetic arrest, near the charged-rod structural glass” section). At these low ionic strengths, the perpendicular diffusion of DNA rods is even coupled to an enhanced twist effect for higher DNA rod concentrations. Here, the parallel motion of DNA rods at higher ionic strengths is greatly hindered, which can be seen in the freezing of random nematic domains.
(II) There are asymmetric transition lines of N–N* occurs in the phase diagram, above the upper binodal line of I–N transition and below the X-pattern, observed non-monotonic increase as a function of the ionic strength: Below the critical ionic strength, pronounced chirality expands as the fd concentration increases. Such asymmetric phase behaviors are clearly visible in the chiral-nematic N* phase and two other chiral mesophases (of X-pattern and helical domains), while as the reorientations of aligned planar nematic phase at higher ionic strengths. Furthermore, this also occurs in the lower-binodal, as N–N* and I–N transition lines appear (see the most left of Fig. 2, at 0.032 mM salt).
(III) The location of a structural glass is observed at high DNA-rod concentrations at the low ionic strength (of 0.16–0.8 mM salts), carrying on the initial caging of fd particles resulting from long-range electrostatic repulsion through thick electric double layers^30,32^. The structural glass is not only isolated within the helical domains but also bounded facing the planar-nematic phase at high ionic strengths (see the red lines in Fig. 2). Different phase boundaries of chiral DNA virus rod orientations are depicted at high rod concentrations, in the tightly packed helical domain, as well surrounded in X-pattern broadly at lower ionic strength. The upper phase boundary of X-pattern is even modulated by the fluctuation that exceeds the critical ionic strength of 1–2 mM Tris/HCl buffer solution (shown as a dashed pink line). Most orientation textures exist only below the critical ionic strength (of 1–2 mM). At the lowest ionic strength (of 0.032 mM salt), further extended chiral-nematic N* and chiral-mesophases appear in a larger range of concentrations. Such orientations textures are not present at high ionic strengths (above 4 mM).
(IV) There is a critical ionic strength of 1–2 mM, in the phase diagram in terms of the intermediately high DNA rod concentrations. By lowering the DNA virus fd rod concentration, below the structural glass, the chiral-nematic N* phase and X-pattern are extended to a larger range of the DNA rod concentrations. These most pronounced chiral phases are demonstrated at the lowest ionic strength (of 0.032 mM), with their depolarized optical morphologies of N* phase, X-pattern and helical domains. Note that here, the microscopic views of helical domains $H_{D}$ with their slow dynamics are provided in the supplementary movie data from Movies 6 and 7 (in Fig. 2), which are quite similar to the larger scale of the chiral-nematic N* phase. However, in contrast, this is not the case for higher ionic strengths above 1 mM salt, which only have an isotropic-nematic phase line.
(V) The self-similarity of pattern formation between the chiral-nematic N* phase and helical domains $H_{D}$ is a quite direct visualization of the replica, in terms of their orientation textures. This also occurs kinetically arrested orientations for few locations of LTKAs among crowded charged DNA rods for a given ionic strength. It is expected that the order parameter will change with the effective diameter of the charged rod, depending on the ionic strength and the effective volume fraction for the concentration. To validate further near the structural glass, few LTKAs are also analyzed via image-time correlation to depict mesoscopic relaxations. The LTKAs are extracted and shown in Movies 1–5. Details of the principle for an image-time correlation can be found in Ref.^34^. At high concentrations of DNA rods for varying the low ionic strengths, the time-lapsed optical morphologies are shown in Fig. 3, and the results of image-time correlations are provided in Fig. 4B for the longer duration time. Figure 4A is the zoom-out view of the correlation functions for different LTKAs.

## Long-time kinetic arrest, near the charged-rod structural glass

The location of structural glass (of low ionic strength, 0.16–0.8 mM salt) is bounded in the equilibrium phase diagram, such that it faces two different phase boundaries of chiral DNA virus rod orientations: One is the tightly packed helical domain towards lower ionic strengths, and the other is a nematic phase at higher ionic strengths (see the top of a phase map in Fig. 2). The visible initial caging of the structural glass is the result of effective microscopic dynamics of orientations realized by different ionic strengths for a given DNA rod concentration. Apparently, such different phases are observed as the helical domains (at a lower ionic strength of 0.032 mM) and the nematic phase (at a higher ionic strength of 0.8–2 mM). Note that near the glass-state, the appearance of voids is present, seen in the LTKA movies. Slow kinetic arrests are typically shown for both structural glass (for 300 h) and chiral mesophases (of 300–500 h). Moreover, enormously slow waiting time kinetics (of 3 months) are shown in the LTKA at 0.8 mM: The phenomenological observations of relaxation on the optical morphology are analyzed by image-time correlation to quantify the decay rates of long-time varied orientation textures.

Image-time correlation functions can be obtained from the flexible time traces of the transmitted intensity through crossed polarizers and reconstructed as time-stamped images, such that the average value of the overall intensity can be distinguished by the single pixel intensity. Then image-time correlation function is defined as:

3 $$\begin{aligned} C_{V}(t)\;=\;\frac{<(I(t)-<I(t)\!>)\;(I(0)-<I(0)\!>)>}{<(I(0)-<I(0)>)^{2}>}\;, \end{aligned}$$

where the brackets $<\cdots>$ denote the average of all CCD camera pixels for the field of views (as $300 \times 300$ pixels). Details of an image-time correlation spectroscopy can be found in ref.^34^. Long-time correlations are shown for several LTKAs as glass (in 0.16–0.8 mM), X-pattern (in 0.5 mM), the mixed state of X-pattern and helical domains (in 0.08 mM), and N–N-star (in 2 mM) in Fig. 4. Much slower decays are shown in the case of the glass state, in LTKA12 and LTKA23, as compared to LTKA1 and LTKA2, with X-pattern and mixed states of helical domains (in 0.08 mM). LTKA1, LTKA2, and LTKA3 decay to below a value of 0.25 in the correlations. Further discussion on the divergence of a relaxation time and the exponent of a correlation length will be addressed in a separate work, to implement the indicative of RSBs, among interacting charged DNA rods for both helical domains and X-patterns that are stable at high concentrations.

## Salt-dependent effective interactions and microscopic relaxations

As it turns out, relaxation slows down substantially at the chiral-nematic N* phase, while the nematic phase undergoes faster relaxation. It is also essential that a critical ionic strength (of 1–2 mM) is observed as a direct consequence of differences in microscopic relaxations between the salt concentrations. Thus, different phase behaviors (for a given DNA virus suspension) are clearly demonstrated in the phase diagram between lower and higher ionic s

**Figure 5:**
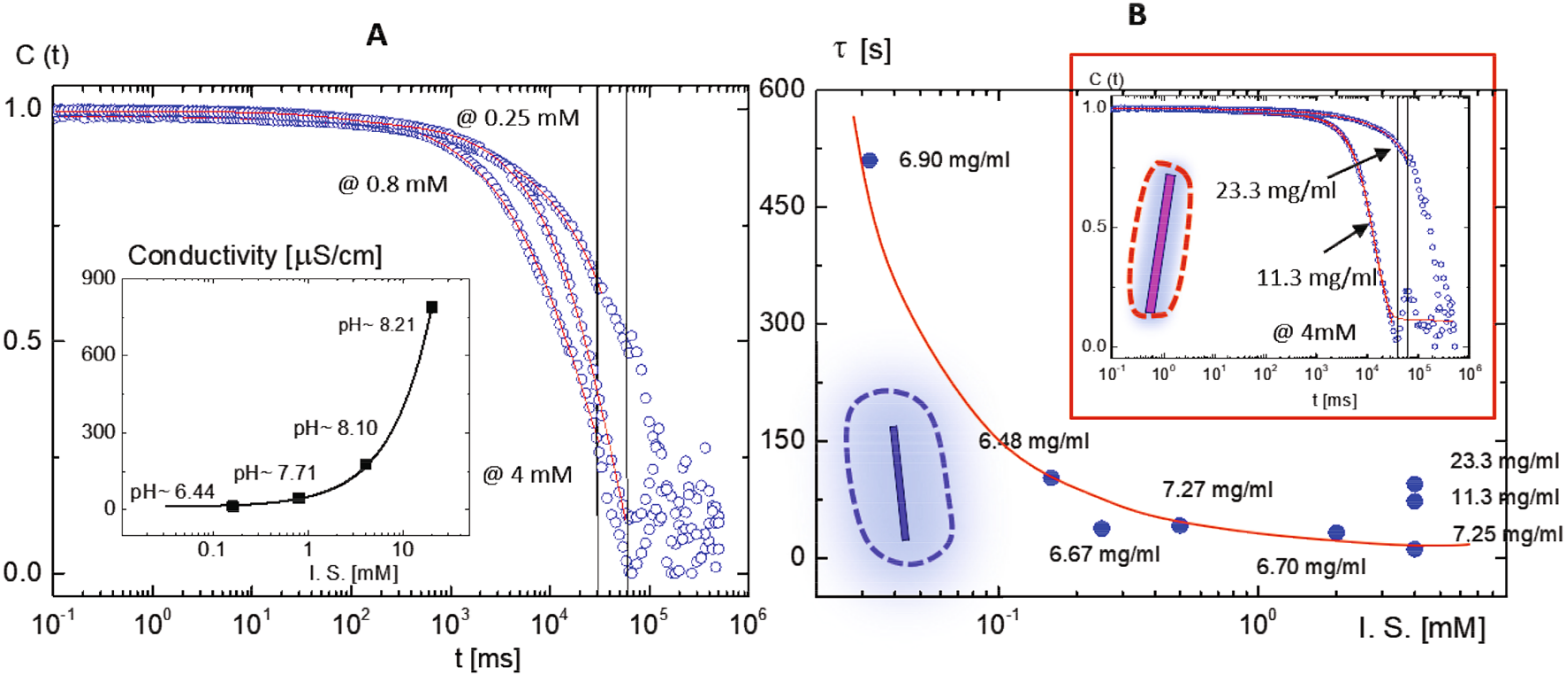
The microscopic relaxations of charged DNA virus (fd) suspensions at varying ionic strengths, measured by small-angle dynamic light scattering: (**A**) The comparison of normalized intensity auto-correlation functions of a fixed fd concentration (of 6.85 mg/ml) for different ionic strengths of 0.25 mM, 0.8 mM, and 4 mM salt. Here, the red lines are the best fit for the reliable data, avoiding the longer-term noise. The red arrow indicates a decrease of ionic strength as well as an increase of electric double layers surrounding the charged DNA rods. The inset shows the conductivity and pH value measured as a function of the ionic strength. Conductivity increases exponentially above 4 mM salt. (**B**) Microscopic relaxations, measured for different fd concentrations as a function of ionic strength: Much slower relaxation times are obtained below 1 mM for the similarly low concentration (for the average of 7 mg/ml). The inset in (**B**) is the comparison of normalized intensity auto-correlation functions for two high fd concentrations at a high ionic strength of 4 mM salt.

trengths than the critical ionic strength (of 1–2 mM salt), which seem to be observed by the salt-dependent microscopic dynamics of collective thermal fluctuations that are sufficiently coupled to their orientations.

The precise description of concentration-dependent orientation textures has difficulty in distinguishing between the torque and twist rates in the microscopic state. Furthermore, in the mean field approach, the pressure tensor is isotropic for an untwisted nematic, however it becomes non-zero when there is a coupling of the surface pressure propagating to the director fluctuations^35,36^. Thus, as a rule of thumb, the rotational field acting on a system is estimated by the strength of torque as compared to the twist. Another difficulty of determining the orientations of a given rod concentration lies in the curvature-elasticity of the bulk chiral (or helical) domains, which becomes complicated in the case of charged hard-rod interactions via chemical potential in the equilibrium. For the sake of simplicity in this case, it is accepted that the nematic domains are large compared to the size of single charged DNA fd rod for the microscopic dynamics. A major consequence of the difference between high and low ionic strengths of charged chiral rod-DNA solutions (like here in fd virus suspensions at varying ionic strengths) is that a variable degree of the dissociation of condensed ions from surrounding fd-coat proteins is then used.

At a high ionic strength (1–2 mM salt) for low concentrations, the nematic state is unstable against the isotropic phase, and the effective concentration is estimated to be lower than the lower binodal concentration, in the vicinity of the I–N coexistent phase. Thus, the effective diameter by relating a dissociation constant is addressed in a previous section (see Eqs. (1), (2)). Equations of motion for the orientation order parameter can be also derived to determine the location of isotropic-nematic phase boundaries^31^. In this work, salt-dependent microscopic relaxation behaviors of the collective dynamics are measured independently by dynamic light scattering for thermal fluctuations of charged DNA virus fd rod suspensions for a given low scattering wave vector of $q \sim 1.8\, \mu {\text {m}}^{-1}$, corresponding to the inverse of a persistent length of DNA rod. Figure 5A shows the relaxation behaviors of a similar range of fd concentrations (about 6.85 mg/ml) for different ionic strengths.

Note that the relaxation times of these two higher concentrations are faster than that of the lower fd concentrations at the ionic strengths (of 0.25 mM, 0.5 mM, and 2 mM salt) shown in Fig. 5A. The comparison of different ionic strengths for the similar range of fd concentration (of 6.85 mg/ml) is given in Fig. 5B, where the normalized intensity auto-correlation functions are fitted to the reliable longer-term window (of 20–30 s). The inset of Fig. 5B shows the comparison of two higher concentrations at a high ionic strength (of 4 mM salt). A slower relaxation time is observed in the intensity auto-correlation function at a higher fd concentration (of 23.3 mg/ml).The relaxation time is increased as the ionic strength decreases, as indicated by the direction of the red arrow. The conductivity is substantially increased above the ionic strength of 0.8 mM at different ionic strengths in the inset of Fig. 5B, indicating that slower relaxation at higher ionic strength is directly influenced by the crowding effects of DNA rods. This then further explains the difference in phase behaviors in Fig. 2: Above the critical ionic strength (of 1–2 mM), the conducting current increases along the longer axis of the thin double layer of DNA virus rods. However, at lower ionic strengths below 1 mM salt, slower microscopic dynamics are dominant as a result of the perpendicular motions of charged DNA rods. This dramatic increase of microscopic relaxation time at the lowest ionic strength (of 0.032 mM salt) is also confirmed by the existing “soft-mode” of charged DNA virus rods, where the twist free energy contribution is coupled to the slower rotational diffusion of charged fd rods at the lowest ionic strength (of 0.032 mM salt)^17^.

## Self-similarity of broken symmetries in 3D helicity and chiral-nematic texture

**Figure 6:**
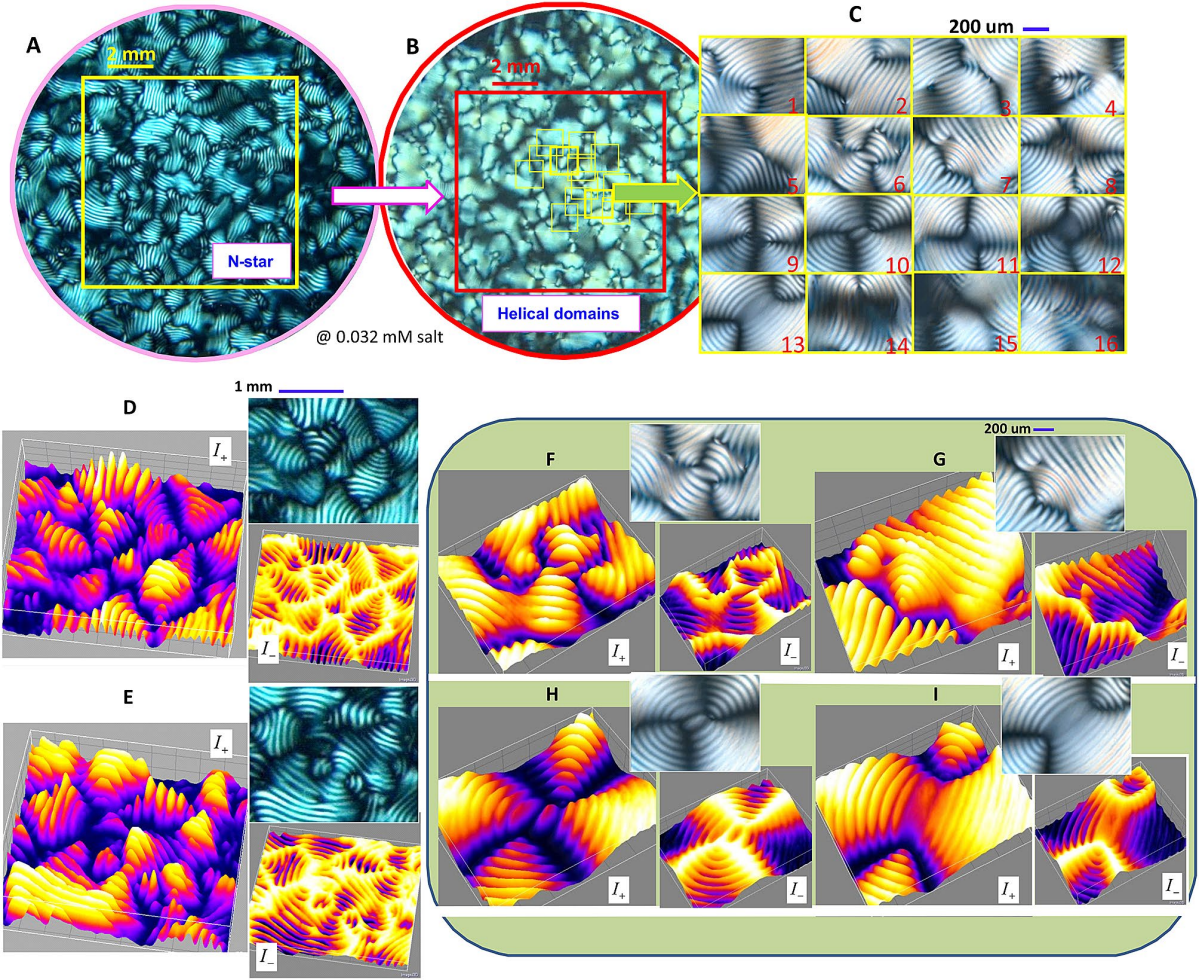
The replica (or self-similarity) of equilibrium states of chiral patterns of N-star (N*) phase: (**A**) and the helical domains (**B**,**C**) at an ionic strength of 0.032 mM salt, with depolarized optical morphologies: The corresponding optical morphologies are enhanced with positive and negative 3D intensity maps of $I_{+}$, and $I_{-}$ for the local view of the N-star (N*) phase (**D,E**) and the varieties of helical domains (**F**–**I**) respectively, by a software version ImageJ 1.52i in the URL link https://imagej.nih.gov/ij/. Note that cavity current loops are visible in both panels of (**G**,**H**), at the center of helical domains, which may also be related to the occasional voids in the phase diagram, as the resultant LTKA1 that can be seen in the Supplementary Data Movie 1.

Since the DNA virus (fd rod) particles are intrinsically birefringent, their orientation textures are used as a direct measure of the concentration-dependent distinguishable orientations in bulk: Under the crossed polarizers, the light regions indicate more aligned phases of DNA rods in average orientations, while the black regions are isotropic states for their random orientations. Various optical morphologies are shown for a lower ionic strength (0.032 mM salt) in Fig. 6. Also, somewhat different optical contrasts of neighboring chiral-nematic domains are observed, depending on the low-ionic strength: At a lower ionic strength (of 0.032 mM), rather uniform color contrast is observed in the polarization of the chiral-nematic N* phase, as compared to two different hues (blue and green colors) of the N* phase at a higher ionic strength (of 0.16 mM). The comparison of depolarized optical morphologies of the N* phase and helical domains are seen in Fig. 6A–C respectively. The reason for such delicate optical contrasts of depolarized morphologies is related to the optical path for transmitted light passing through the 3D bulk orientation textures. It shows that higher contrast appears at an ionic strength of 0.16–0.8 mM salt, where the structural glasses are found in the phase diagram. This suggests that the role of domain boundaries cannot be negligible even for minor difference in the orientations of neighboring chiral-nematic and N-star (N*) orientation textures, which will be discussed in the following section.

**Figure 7:**
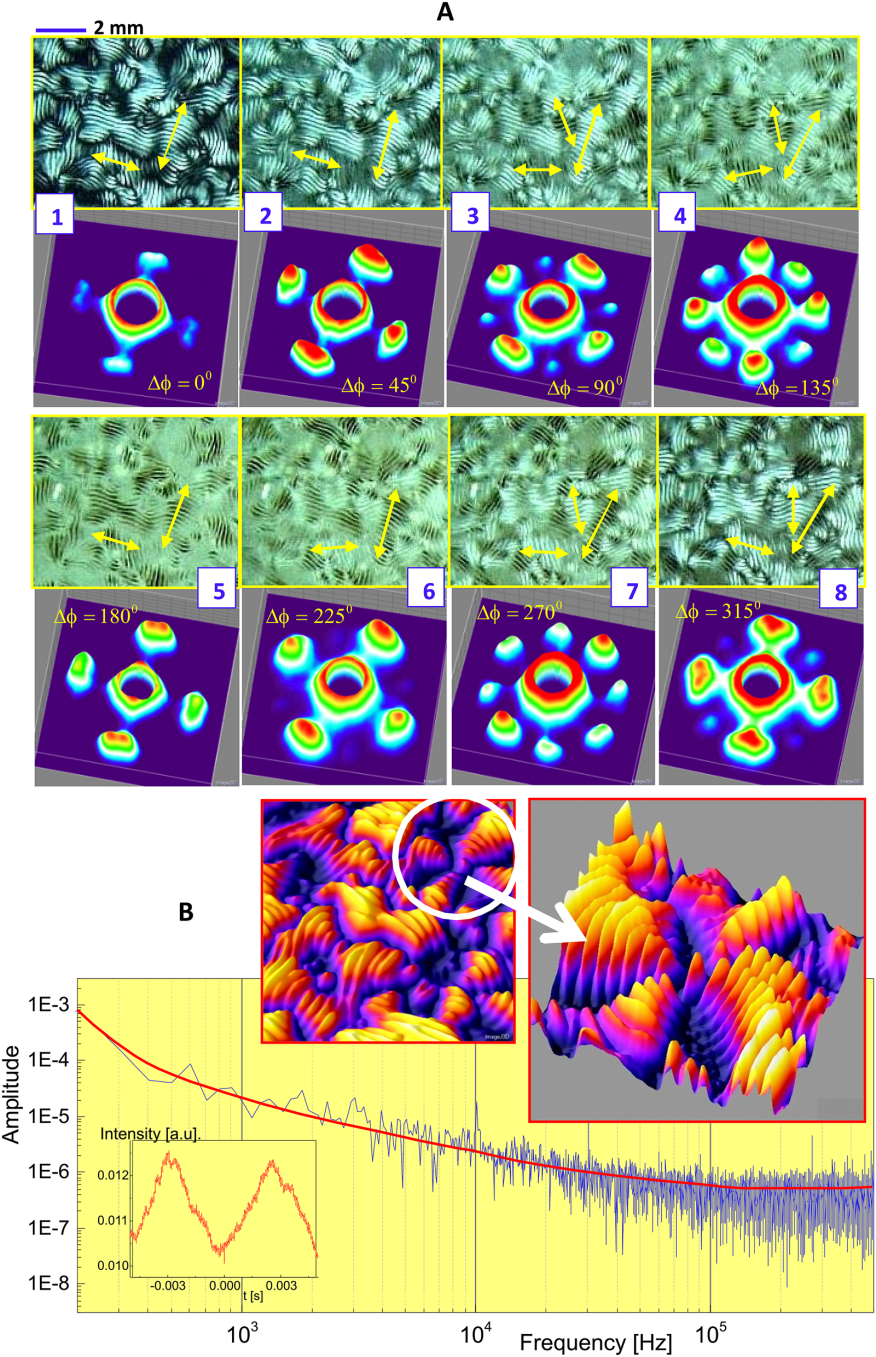
Optical activity of crowded charged DNA viruses (fd) in the N-star (N*) phase at a low ionic strength of 0.032 mM salt: The rotational sequence of depolarized optical morphology is shown in intervals of $\pi /4$ by rotating the polarization (**A**), with the corresponding orientation distribution in Fourier space. The directions of local helicity axes are indicated by the yellow arrows. Eight intensity lobes are shown for equally distributed at an angle of $270^\circ$ (of the panel 7 in (**A**)). (**B**): The actual optical activity of a chiral-nematic phase, measured by the Second Harmonic Generation (SHG) intensity, where the amplitude of a low frequency signal-to-noise ratio is detected as being small, but not negligible. 3D intensity maps are obtained by a software version ImageJ 1.52i in the URL link https://imagej.nih.gov/ij/.

The details of self-similarity with depolarized optical morphology are provided for a chiral-nematic phase and the localized (positive $I_{+}$, and negative $I_{-}$) views in 3D intensity maps in Fig. 6D,E, obtained by the ImageJ software. Depolarized morphologies of tightly packed helical domains are also shown in Fig. 6F–I, with the various types of defect lines. These 3D intensity maps visualize directly the orientation textures formed in bulk elastic deformations. Here, the intensity maps are of use in distinguishing both-side (top/bottom) substrates and for reading the actual intensity pixel values in region of interests (ROIs): For instance, the black disclination lines are clearly depicted as the backbones in negative ($I_{-}$) intensity maps of the depolarized morphology. In the positive ($I_{+}$) 3D intensity maps, the spatial gradients are captured clearly in the individual bright fingerprint (or stripe) textures among the neighboring ones of intensity profiles. Similar trends of intensity maps are shown for the smaller pitch length (~ 100 µm) in helical domains, and likewise larger pitch lengths (~ 100–200 µm) of chiral-nematic textures are shown as the replica in different length scales.

To evaluate the local orientations of a chiral-nematic or N-star (or N*) phase, the angular dependence of an equilibrated chiral-nematic texture (for an fd concentration of 7.0 mg/ml at a low ionic strength of 0.032 mM) is probed by rotating the polarization in intervals of $\pi /4$. The sequential morphologies for local orientations of chiral-nematic textures, including the domain–domain boundaries are, provided with the corresponding orientation distribution and mapped onto the Fourier space in Fig. 7A. Here, the local helical axes of each chiral-nematic texture are indicted as yellow arrows, perpendicular to the stripe patterns that twist among neighboring ones. These chiral-nematic domains exhibit very slowly varying dynamics in the space modulated between neighboring defect lines (provided as Movies 6, 7 in Fig. 2). The depolarized morphology and the corresponding Fourier transforms are labeled with the numbers 1,2,...,8 in increasing intervals of $45^{\circ }$. An optical pitch length of 200 $\mu$m (of a chiral-nematic phase) is shown among twisted interfaced lines that undergo continuous changes when a polarizer is rotated. Optical activity is also measured independently by means of Second Harmonic Generation (SHG) intensity in Fig. 7B, where the amplitude of a low-frequency signal-to-noise ratio is indeed detected to be small in local orientations, but not negligible, due to a non-zero third harmonic susceptibility at a low frequency regime. Thus, it agrees with other chiral materials for local anisotropic orientation that is expected to occur in case of a polarization of nonlinear susceptibility greater than zero^37,38^, as well as for the intrinsic chirality of protein helices and collagen fibrils^39,40^. In Fig. 7B, the non-zero amplitude is observed at a frequency domain (below about 10 kHz), which is responsible for locally oriented chiral DNA virus fd rods and the anisotropy, induced by an increase of fd-rod concentration. Therefore, one should note that here, thick electric double layer–layer interaction-induced self-assembly has a completely different origin with the thin electric double layers that are mostly for dielectrically polarizable materials by a strong surface anchoring on a substrate or by adding chiral dopants.

For the fixed lowest ionic strength (of a 0.032 mM salt), interestingly, the structural glass is not observed. Instead helical domains $H_{D}$ persist for given high DNA rod concentrations. The replica occurred in the equilibrium phases of charged fibrous viruses (fd), being as the chiral-nematic N* phase, and a hierarchical chiral mesophase of the helical domains, above the nematic-isotropic coexistence concentration. However, between them, RSB exist at an intermediate concentration, a chiral-mesophases of X-pattern, driven by the randomized and fast (propagating) mechanical kink of orientations for interacting long-range electrostatic interactions in bulk elastic medium. Also, the slow process of RSB for a given low ionic strength is of interest, as the DNA-rod concentration increases, in terms of the formation of a cavity current loop (a notion introduced by Onsager in 1949). Similar features are clearly shown in the circulation of orientation textures in LTKA2 for the X-pattern (see Movie 3) between the chiral-nematic phase and helical domains. This is then related to the fact of either continuous change of the pitch variances or the internal stress release between the twisted boundaries of orientation textures. The possible scenario of local orientations with other neighboring charged DNA-rods in the X-pattern is to be discussed separately, where the cavity current loop may be related to cancellation of opposite directions in their alignments.

**Figure 8:**
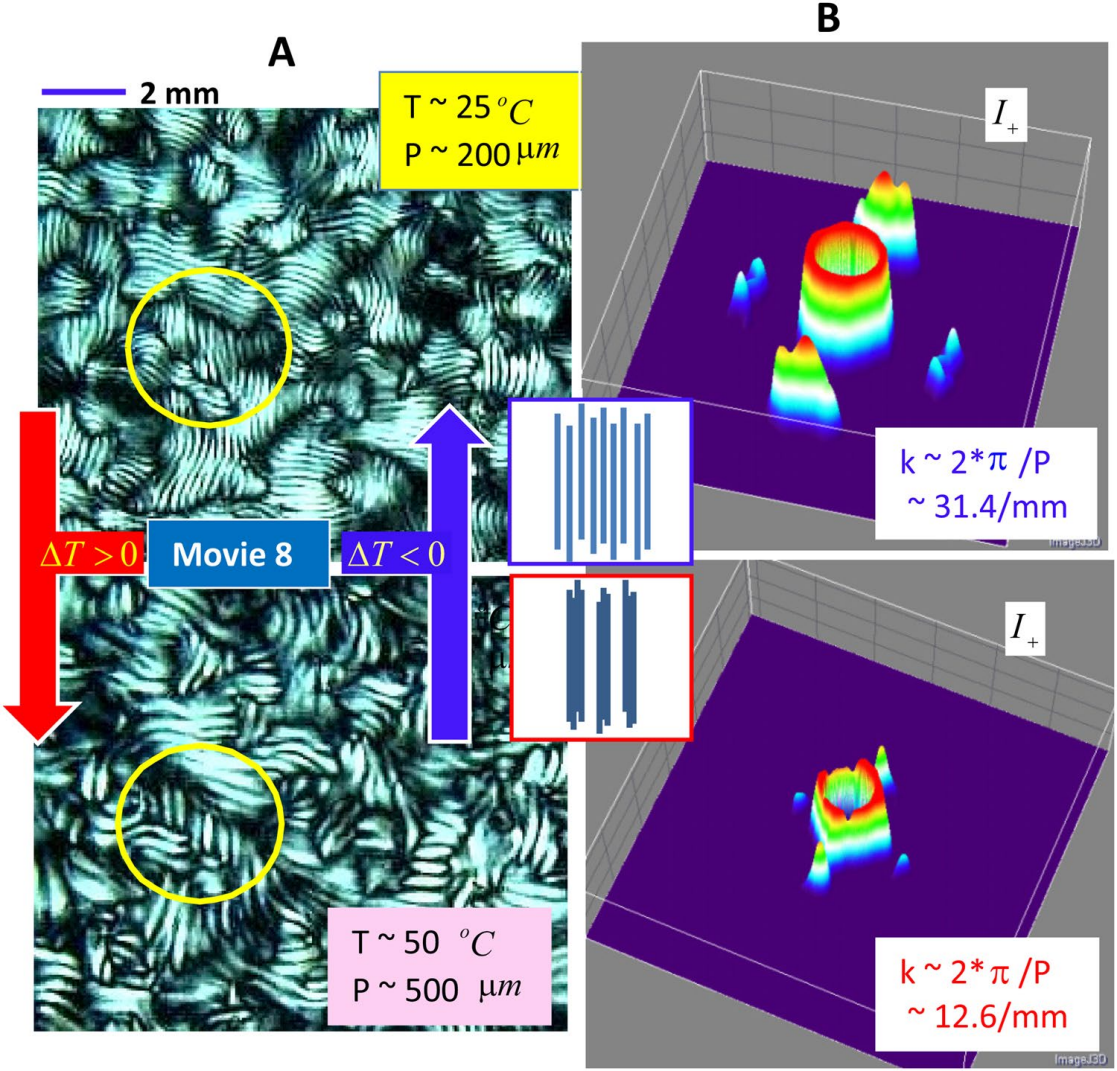
Thermal stability of chiral-nematic, N-star (N*) orientation texture: Thermal response of the optical pitch variances of a chiral-nematic N-star (N*) phase of a low ionic strength of 0.032 mM salt in real space (**A**), and the corresponding Fourier transforms in 3D intensity profiles (6) The temperature swap occurs for two different temperatures of 25 $^{\circ }$C (upper) and 50$\,^{\circ }$C (lower), respectively, by a 3D intensity map obtained by a software version ImageJ 1.52i in the URL link https://imagej.nih.gov/ij/. Movie 8 shows supplementary data for the reversible pitch length between 200 $\mu$m (at room temperature, 25 $^{\circ }$C) and 500 $\mu$m, when the temperature is increased to 50 $^{\circ }$C. The temperature increases to the higher value over the first half of the duration and decreases to the lower value over the other half.

**Figure 9:**
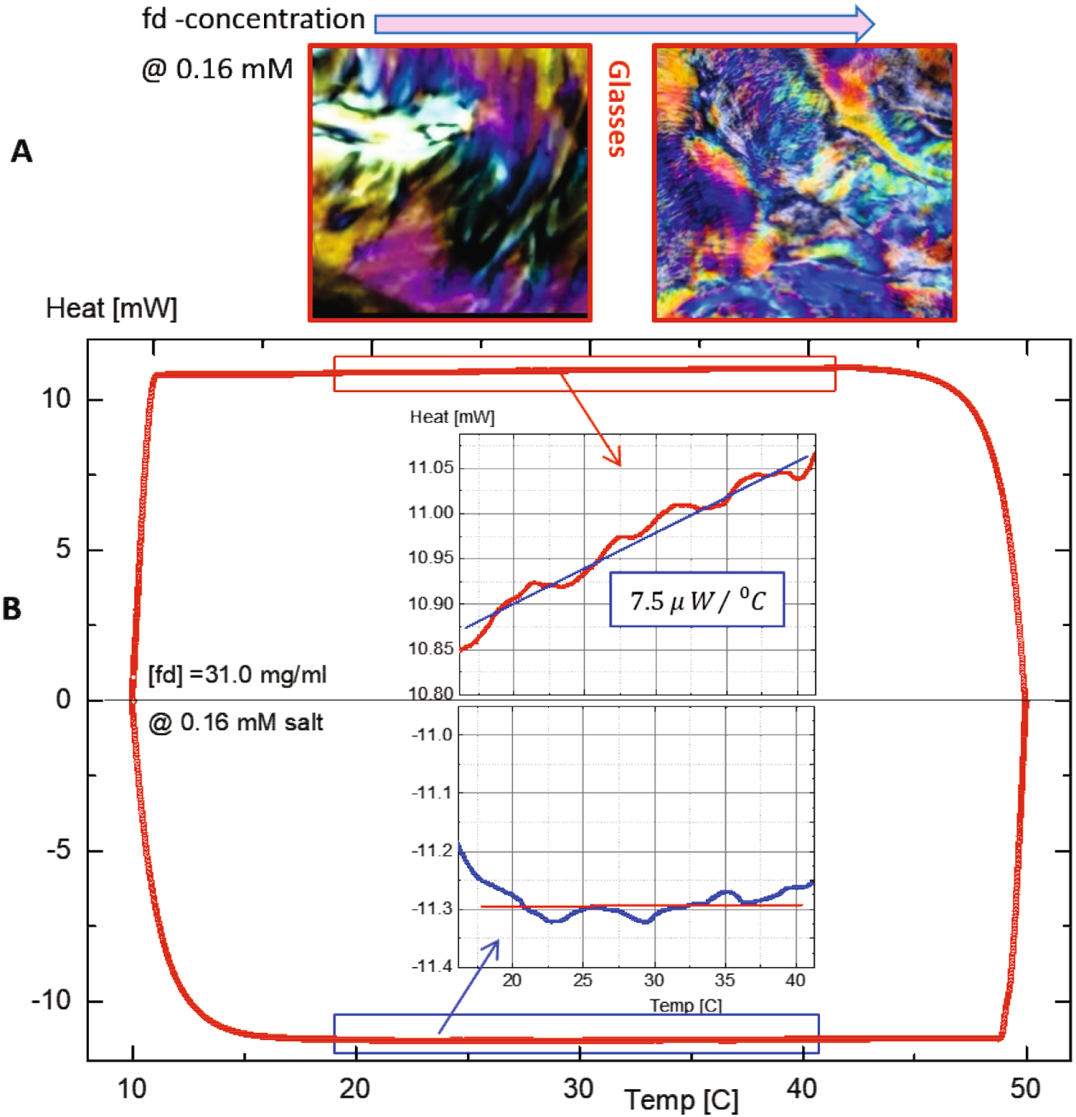
(**A**) Different optical contrasts of the glass state at a low ionic strength of 0.16 mM, where more colorful birefringence is evident as the concentration increases. (**B**) Athermal specific heats of glass state are measured by the heat flow of a high concentration for the glass concentration (of 31 mg/ml) at a low ionic strength of 0.16 mM salt by differential scanning calorimetry (DSC). The insets show the specific heats with the different response for the heating (top) versus the cooling (bottom) process respectively. The constant value of specific heat during cooling may explain the steadiness of the thermal stability, for both the glass states and the chiral-nematic orientation textures in the equilibrium phases.

## Thermal stability of reversible optical pitch and abnormal specific heat of glass

Thermal stability of DNA rod suspensions is important for maintaining the phase behaviors. The temperature responses of pitch variance are probed independently in the stable chiral-nematic N*phase at a lower ionic strength (of 0.032 mM salt), as shown in Fig. 8A. A commercially available temperature oven (TSA02i-mK1000, Instec HCS60 Inverted Microscope Thermal Stage) is used for the fd concentration of 5.4 mg/ml at a lower ionic strength of 0.032 mM. The initial state is at a room temperature of 25 $^{\circ }$C, which is slowly increased to a higher temperature of 50 $^{\circ }$C with a ramp rate of 0.2 $^{\circ }$C/min. The thermally reversible correlation lengths of chiral-nematic pitch lengths are provided. The final state is reached in reverse by cooling to room temperature at the same rate. By cooling the sample, the reversible pitch length is observed (see the Supplementary Movie 8). At room temperature (of 25 $^{\circ }$C), in the top image of Fig. 8A, the optical pitch *P* is measured as 200 $\mu$m, while at 50 $^{\circ }$C (in the bottom image of Fig. 8A), the pitch length increases to 500 $\mu$m. By increasing the temperature, shorter Fourier spacing is observed with an increased pitch length. The shift of Fourier peaks to smaller spacing confirms that the pitch increases (or broadens) on increasing the temperature (see Fig. 8B).

In addition, the high-concentration glass states maintain their initial caging, which is shown as the colorful birefringence in Fig. 9A. A wider spectrum of colors with smaller sizes of randomized domains appear when the concentration of the glass state increases. At such higher DNA rod concentrations near the glass states, a wide range of colors also appeared in the initial caging (see the images of Fig. 9A). This is why the case is not yet fully exploited, which may require a careful estimation of the effective “twist power” for orientation textures or the given helical domains. Also, the heat flows are measured by DSC (Differential Scanning Calorimetry) for a high-rod concentration glass state (of 31.0 mg/ml) at a given ionic strength (of 0.032 mM) in Fig. 9B. Different responses of specific heat are detected, between a heating (top inset) and a cooling (bottom inset) process (in the inset of Fig. 9B).

Surprisingly, there is no variation of the specific heat in the cooling process, meaning that local thermal access of mobile ions is not affected at all: By decreasing the temperature, a constant specific heat (bottom inset of Fig. 9B) is observed, while the slower increase of heat flow is still measured in a heating process (top inset of Fig. 9B). This abnormal specific heat, found when the temperature is decreasing in particular, may also show the reversible pitch variances, seen in above Fig. 8, of the chiral-nematic textures. It is then indicated that the release of condensed ions for charged DNA viruses (fd) is sufficiently initiated by the degree of accessible mobile ions at a higher temperature for thicker electric double layers to result. In contrast, the finite pitch length is sustained by the cooling process at a lower temperature. Therefore, the microscopic relaxation cooperates to form the possible thermodynamically stable chiral pitches and the reproducible correlation length (seen in Supplementary Data Movie 8) in case of moderate temperature changes. This observation can be useful for facilitating further exploration of abnormal specific heat in the cooling and heating process to compare other types of interacting charged rods in thermodynamic systems.

**Figure 10:**
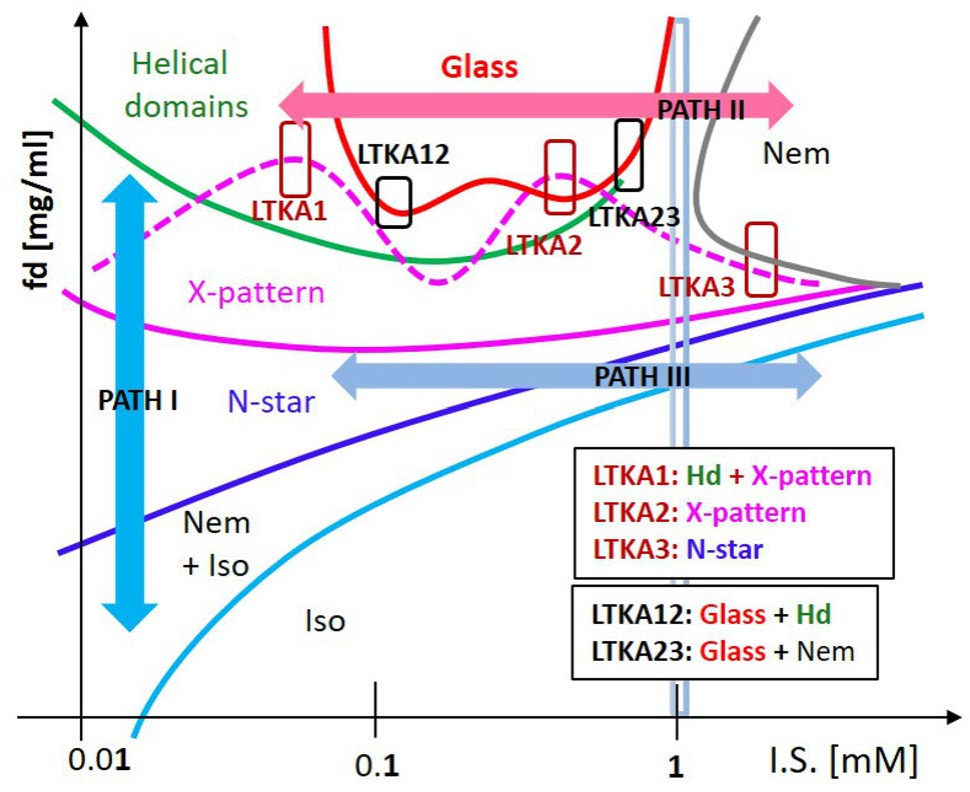
Simplified equilibrium phase diagram of several phases for chiral-nematic mesophases, chiral-mesophases, and the glass states, as well as with different phase boundaries. The dynamic pathways are shown as Path I, Path II, and Path III in logarithmic scales of ionic strength: The phases of each LTKA are expressed in the arrangement of orientation textures in the phase diagram (see also Fig. 2).

## Discussion

The equilibrium phase diagram of charged DNA virus chiral rods above isotropic-nematic coexistence concentration is found to be rich in the phase behaviors that are observed in the plane of DNA rod concentration and ionic strength. Rather complicated phases and their neighboring phase boundaries are observed with the equilibrium or metastable phases (LTKAs), as the resultant macromolecular twist orders in randomized 3D bulk states occur. The salt-dependent effective interactions of charged chiral DNA rods are illustrated in the simplified phase diagram of Fig. 10, where particularly three dynamic pathways for approaching each phase are highlighted. Self-similar organizations, as the replicas of twist orientation orders, persist as the stable phase in different length scales; a few times larger than the correlation length of chiral-nematic, N* phase optical pitch (around 200 $\mu$m) at lower concentrations, while a relatively small pitch (of 20 $\mu$m) in tightly packed helical domains with twisted interfaced boundaries at higher concentrations. Some indications of the broken symmetries of orientation textures are observed in LTKAs, which are analyzed by image-time correlation in the phase diagram. At the critical ionic strength (of 1–2 mM) for the given DNA rod concentration, different levels of microscopic diffusivity are caused by the perpendicular motion and increased by lowering the ionic strength. This then seems to contribute to the pronounced coupled motion of local helicity that is responsible for stabilizing the bulk chiral mesophases. To distinguish the phase behaviors, dynamic pathways of PATH I, II, and III are drawn and summarized as follows:

- *PATH I* The dynamic pathway PATH I is shown as a chiral-nematic N-star (N*) phase, X-pattern and helical domain $H_{D}$, as an increase of the DNA rod concentration for a given low ionic strength. At the lowest ionic strength (of 0.032 mM), greatly extended regions of the chiral-nematic N* phase and the hierarchical chiral-mesophases (of X-pattern and helical domains) are observed, as the DNA rod concentration increases. This is possible due to the easy access of the released condensed ions via thick electric double layers at such low ionic strength, leading to the effectively larger Debye screening length.
- *PATH II* Across the critical ionic strength (of 1–2 mM), by lowering the ionic strength, at high DNA-rod concentrations, the nematic state undergoes extremely slowed relaxation to reach a structural glass that bears somewhat transient features of the initial caging, local stress release, and long-time metastable states (see the PATH II). The glass of charged fd rods is found at the low ionic strength (of 0.16–0.8 mM salt), located between the helical domains and the nematic-phase. Near the glasses, long-time kinetic arrests (LTKAs) are analyzed via image-time correlation for the characteristic decays of their initial caging and final states.
- *PATH III* Below the intermediate DNA rod concentration and the critical ionic strength (below 1 mM), a stable chiral-nematic N*-phase is formed above the isotopic-nematic coexistence concentration, while above this concentration, a nematic state is dominant, in PATH III. Here, notably asymmetric phase boundary lines are related to the effective interaction of charged fd rods, proved by the microscopic dynamics of collective thermal fluctuations that are coupled to orientations: For lower ionic strengths, the substantially slowed dynamics of a chiral-nematic N* phase are related to the perpendicular component of translations. In contrast, parallel motion is preferred in the nematic phase at high ionic strength, which is also assisted by the increased conductivity of the fd rod itself (seen in the inset of Fig. 3B).
- *Between the PATH II and III* The most intriguing observation in the phase diagram is the region where the upper and lower phase boundaries of the X-pattern appear, such that the lower one is rather distinctive, compared to the upper phase boundary, which fluctuates as a function of ionic strength. This feature is now designated between PATH II and PATHIII. Typically, there is a mechanical kink at the lower border line of the X-pattern; however, the continuous changes occur at the upper border lines. The location of the X-pattern is between the chiral-nematic phase and the helical domains, where the rotational motions are seen at LTKA2 with the supplementary data of Movie 3. The X-pattern can be treated as the RSB, where substantially larger rotational diffusion is achieved at the lowest ionic strength, as well as the translation being mostly hindered. Thus, at high rod concentrations for lower ionic strengths, effectively pronounced slower dynamics are coupled with the concentration, forming the stable chiral-nematic N* phase and X-patterns. The details of the X-pattern will be studied in a separate paper discussing the divergence of optical pitch length and the critical slowing down of local helicity.
- *Below the PATH III* The asymmetric phase boundary lines are found at the N–N-star (N*) transition line above the I–N coexistence concentration. Here, the divergence of chiral-nematic optical pitch length occurs during the approach from the N-star (N*) to the N phase as the ionic strength increases. Also, the reverse will occur, which then also mimics the symmetry breaking of local helicity for charged DNA rods. Therefore, below PATH III, the twist effect at the low ionic strengths is further affected by the enhanced couplings of rotation and translation (in particular, in the perpendicular direction) for the collective motions above the I–N coexistence concentration. Such couplings can be further extended to achieve a minimum coherence length, from the N-star (N*) phase to the X-pattern via electrostatic repulsive forces or torques^17^.

To conclude, the current system shows visible 3D orientation phase behaviors in equilibrium, with several phase boundaries of different pathways for twist-interactions of crowded charged DNA viruses (fd rods) at low ionic strengths. The delicate interplay between the short-range helical core–core interactions and long-range electrostatic interactions leads to both a reduction of optical pitch (from a chiral-nematic N* phase to a helical domain, $H_{d}$), and to hierarchical chirality (in the X-pattern and helical domain, $H_{d}$)). With regards to RSB near the structural glass, both helical-domains and the X-pattern are stable at high concentrations for low ionic strengths, exploited by the appointed long-time kinetic arrests (LTKAs) in the phase diagram. However, at higher ionic strengths, the glass state encounters frozen nematics (at LTKA23). Within the equilibration time for low concentrations, several distinguishable equilibrium orientation textures exist (see PATH I). Finally, thermal stability of the optical pitch length (of a chiral-nematic N* phase) and the abnormal specific heat (of a glass state) are demonstrated. Furthermore, the temperature change between the N* phase and the X-pattern also contributes to validating the reversibility of a transition. In particular, the X-pattern demonstrates cavity current loops (in LTKA2), which are driven by an effective twist potential. The divergence of a relaxation time and the exponent-power of a correlation length among interacting charged DNA rods will be further examined, in subsequent work, as a new type of a glass, so-called as a “chiral-glass”. Therefore, the indicative of RSBs near the structural glass for both helical domains and the X-pattern are stable at high concentrations, below the critical ionic strength, relating to the extreme slowing down of the local helicity for high-concentrations and the chiral-mesophase (of helical-domains and the X-pattern). In addition, whether there is any effective temperature relating to the dissociation/association of condensed and mobile ions for DNA rods in the N–N*, and N*–X pattern transitions at low temperatures, is another topic that is worthy of study.

We hope that the present study leads to future theoretical studies involving the various observed phenomena in lyotropic systems of charged rod-like colloids, and possibly opens pathways that would be helpful in the construction of DNA-based materials. These phenomena include the formation of different morphologies in chiral mesophases, depending on concentration and ionic strength, the nature of soft glass transition of rod-like objects, including long-time kinetic arrest, and the kinetics of phase transformations. The understanding of such phenomena may contribute to the role played by generic interactions between DNAs in living systems.

## Methods

*The morphology of orientation textures* is collected by varying ionic strength and DNA rod concentrations, and by building up the phase diagram under a depolarized light in a commercially available flat cylindrical Hellma cell, with a circular diameter of 20 mm and a thickness of 1 mm. The typical sample amount is about 380 $\mu$l. Also, image-time correlation spectroscopy is conducted to extract the mesoscopic decay rates for a few selected high concentrations of LTKAs near the glass state for varying ionic strengths.

*Image-time correlation functions* can be obtained from the time traces of the transmitted intensity through crossed polarizers and are shown with the CCD camera for a single pixel. The collection of raw time-lapse images are then reconstructed as time-stamped images, such that each frame is processed for the average value of the overall intensity, meaning that black and white pixel values are different from the average gray pixel intensity. The image-time correlation function is defined such that the single image in a time frame is used to construct an image correlation function, where the region of interest is $300 \times 300$ pixels. The above definition of the image time correlation function is reminiscent of the intensity correlation function as obtained from dynamic light scattering, except that the intensity here is not a scattered intensity, but instead the transmitted intensity through the two crossed polarizers. Here, ImageJ software is used in Figs. 2, 3, 4, 6, 7 and 8.

*The nonlinearity of optical activities* is checked by a second harmonic generation (SHG), at higher field strength for the narrower gap distance: The ITO coated glass cells with thickness 5 $\mu$m are used for optical studies with the Axio Imager A.1 polarizing microscope (Carl Zeiss GmbH). Measurements of the second harmonic generation (SHG) are performed in cells by using Nd: YAG laser (1064 nm, 10 ns pulse duration and 10 Hz repetition rate). The fundamental light beam is incident at an angle of 45° of the normal to the cell. The SHG signal is collected in the transmission direction by a photomultiplier tube (Hamamatsu).

## Supplementary Information

Supplementary Information 1.

Supplementary Information 2.
