# Supplementary material for: Equilibrium phase diagram and thermal responses of charged DNA-virus rod-suspensions at low ionic strengths
Source: Sci Rep. 2021 Feb 10;11:3472. doi: 10.1038/s41598-021-82653-y (PMC7876043; doi:10.1038/s41598-021-82653-y)
Supplement: Supplementary file 1 — Supplementary Information 1. [file 41598_2021_82653_MOESM1_ESM.pdf]

SUPPLEMENTARY MATERIALS

Title: Equilibrium Phase Diagram and Thermal Responses of Charged DNA-virus Rod-Suspensions at Low Ionic Strengths

Auhtor: Kyongok Kang

See the supplementary material of 8 movies:

Movie 1: A speed up movie of long-time kinetic arrest (LTKA1) for the fd-concentration of 14.7 mg/ml and the ionic strength of 0.08 mM, in the equilibrium phase diagram in Fig. 2. Duration is about 10 days.

Movie 2: A speed up movie of long-time kinetic arrest (LTKA12) for the fd-concentration of 13.8 mg/ml and the ionic strength of 0.16 mM, in the equilibrium phase diagram in Fig. 2. Duration is about 45 days.

Movie 3: A speed up movie of long-time kinetic arrest (LTKA2) for the fd-concentration of 14.5 mg/ml and the ionic strength of 0.5 mM, in the equilibrium phase diagram in Fig. 2. Duration is about 10 days.

Movie 4: A speed up movie of long-time kinetic arrest (LTKA23) for the fd-concentration of 14.7 mg/ml and the ionic strength of 0.8 mM, in the equilibrium phase diagram in Fig. 2. Duration is about 90 days.

Movie 5: A speed up movie of long-time kinetic arrest (LTKA3) for the fd-concentration of 7.5 mg/ml and the ionic strength of 2 mM, in the equilibrium phase diagram in Fig. 2. Duration is about 10 days.

Movie 6: A speed up movie of equilibrated helical domains in Fig. 2, within highly packed domain texture, where thermal fluctuation exists in a very slow time variable. The movie consists of 10 min intervals over 53 hours.

Movie 7: A speed up movie of equilibrated helical domains in Fig. 2, within highly packed domain texture, where slowly varying of domain boundaries are present due to the microscopic thermal fluctuations. The movie consists of 10 min intervals over 24 hours.

Movie 8: A speed up movie of thermally reversible, equilibrated chiral-nematic texture, for the fd concentration of 7.0 mg/ml and the ionic strength of 0.032 mM, shown in Fig. 8A. Initial state is at a room temperature (at 25 Celsius) and slowly increased to higher temperature (at 50 Celsius) with a ramp rate of 0. 2 Celsius/min and then cooled to a final state of the room temperature at the same rate. Half of the movie covers the heating and the other half is the cooling process.
